# Imaging of Bone Metastases in Breast Cancer

**DOI:** 10.1053/j.semnuclmed.2022.01.005

**Published:** 2022-02-27

**Authors:** Gary J.R. Cook

**Affiliations:** † Cancer Imaging Department, School of Biomedical Engineering and Imaging Sciences, King’s College London, London, UK; ‡ King’s College London & Guy’s and St Thomas’ PET Centre, St Thomas’ Hospital, London, UK

## Abstract

Bone metastases are a common site of spread in advanced breast cancer and responsible for morbidity and high health care costs. Imaging contributes to staging and response assessment of the skeleton and has been instrumental in guiding patient management for several decades. Historically this has been with radiographs, computed tomography and bone scans. More recently, molecular and hybrid imaging methods have undergone significant development, including the addition of single photon emission computed tomography/computed tomography to the bone scan, positron emission tomography, with bone-specific and tumor-specific tracers, and magnetic resonance imaging with complementary functional diffusion-weighted imaging. These have allowed different aspects of the abnormal biology associated with bone metastases to be explored. There is ability to interrogate the bone microenvironment with bone-specific tracers and cancer cell characteristics with tumor-specific methods that complement morphological appearances on computed tomography or magnetic resonance imaging. Alongside the advent of novel, more effective and nuanced therapies for bone metastases in breast cancer, there is accumulating evidence that the developments in imaging allow more sensitive and specific detection of bone metastases as well as more accurate and earlier assessment of treatment response leading to improvements in patient management.

## Introduction

Bone metastases are common in breast cancer with 50-70% of patients affected at relapse^1,2^ and with 28%-44% show-ing bone-predominant disease.^3,4^ The clinical importance of skeletal metastases is related to skeletal related events (SREs) including pain, pathological fracture, spinal cord compression, hypercalcemia and bone marrow dysfunction; morbidity that may occur in greater than 50% of patients with metastatic breast cancer. Those with bone-predominant disease are more likely to develop complications (80%) compared to mixed bone and visceral metastases (60%) or no bone disease (21%).^5^

Compared to bone metastases from other cancers, breast cancer patients have a better prognosis with median survival of more than 2 years in those with disease confined to the skeleton compared to less than 6 months in those with visceral disease.^3,6^ At the same time, with an increased understanding of underlying pathophysiology of bone metastases and discovery of new targets, more effective therapies have been introduced. Some of these specifically target bone, including the antiresorptive effect of bisphosphonates or inhibition of receptor activator of nuclear factor-*k*B ligand (RANKL) by denosumab.^7^

It is therefore imperative that bone metastases are detected early so that effective treatment can be instigated to minimize SREs and that treatment response can be measured accurately to limit toxicity and enable rapid therapeutic transition to second or third-line treatments in nonresponding patients. These are both areas where imaging can make a major contribution with adoption of novel imaging allowing improved sensitivity and specificity for detection and response assessment compared to historical methods. With molecular imaging techniques there is the ability to target specific aspects of abnormal tumor or bone metabolism related to skeletal metastases.^8–10^

## Pathophysiology of bone metastases applied to imaging

Paget proposed the seed and soil hypothesis whereby metastatic cancer cells are attracted to a favorable bone marrow microenvironment in which they can subsequently grow following a period of dormancy in the metastatic niche.^7,11,12^

In healthy bone there is a balance between bone resorption by osteoclasts and bone formation by osteoblasts to maintain bone integrity. However, in most cancers, an osteolytic metastatic phenotype predominates. Here, parathyroid hormone related protein, derived from cancer cells, stimulates the production of RANKL which is associated with increased osteoclast maturation and bone resorption, overcoming attempts at osteoblastic bone formation and repair.^7,13^ In some cancer types (eg, prostate cancer), an osteoblastic phenotype predominates where cancer cells secrete factors that stimulate aberrant osteoblastic bone formation.^7^ While osteolytic metastases predominate in breast cancer, mixed osteolytic and osteoblastic lesions are frequently seen. Following therapy, osteoblastic repair at metastatic sites may mimic primary osteoblastic tumor induced activity on imaging, making it impossible to differentiate these two processes.

An understanding of the underlying biology of bone metastases is important as these factors may influence the choice of tracer and allows consideration of the strengths and weaknesses of different available imaging methods depending on the clinical question.

Historically, imaging has relied on altered bone morphology to detect and characterize bone metastases with radiographs or computed tomography (CT). However, it is recognized that more than 50% of bone mineral may need to be lost before a lesion becomes visible as an area of lysis/reduced density on radiographs, making this method relatively insensitive. For several decades, the nuclear medicine bone scan, using bone-specific tracers such as ^99m^Tc-methylene diphosphonate (MDP), has shown greater sensitivity than radiographs or CT for detection of bone metastases. Uptake of these bone-specific tracers relies on local increase in blood flow and osteoblastic activity.^14^ This indirect method of detection of metastatic tumor is therefore unable to sensitively detect metastatic lesions in the marrow before an osteoblastic reaction has taken place or in lesions where lysis predominates (eg, myeloma). Specificity for detecting metastatic lesions may also be limited without further morphological imaging as several benign processes can cause focal uptake in the skeleton.^15^ In addition, determining treatment response with bone scans may be impaired as it may not be possible to differentiate progressive osteoblastic disease from reparative osteoblastic repair after successful systemic therapy for several weeks or months. The latter phenomenon is the flare response that has been described following endocrine therapy and chemotherapy in metastatic breast cancer.^16,17^ This phenomenon has also been recognized using ^18^F-fluoride as a bone-specific PET tracer, as ^18^F-fluoride has a similar mechanism of uptake to the ^99m^Tc-labeled diphosphonates.^18^

Methods for imaging increased osteoclast activity have been described for metastatic bone disease in preclinical studies and in other cancers.^19–22^ These have used SPECT and PET labeling of the Arg-Gly-Asp (RGD) motif that binds to the *α*v*β*3 integrin that is highly expressed by osteoclasts and aids attachment of the osteoclast to bone during resorption.^23^ While an attractive method for detecting this aspect of abnormal bone metastasis biology, and potentially acting as a method to monitor response to osteoclast-inhibiting treatments such as bisphosphonates or denosumab, these have not reached sufficient evidence for clinical adoption.

The use of tumor-specific tracers for investigating bone metastases has become more common. These have the potential advantage of detecting abnormal tumor cell metabolism before an osteoblastic reaction has taken place, thereby offering improved sensitivity and a more direct measure of tumor response that is not affected by changes in the bone microenvironment. The increased aerobic glycolysis that occurs in cancer cells, with upregulated glucose transporter (GLUT 1) and hexokinase II expression, is a phenotype shared by most breast cancers and can be sensitively imaged with ^18^F-fluorodeoxyglucose positron emission tomography (^18^F-FDG PET). It has been reported that ^18^F-FDG PET is more sensitive in detecting bone metastases from ductal compared to lobular breast carcinomas, possibly due to differences in cellular density, proliferation rate and GLUT1 expression.^24^ Sclerotic metastases are recognized as being less ^18^F-FDG-avid than lytic metastases. Lobular breast cancer metastases, in particular, are more frequently sclerotic and less ^18^F-FDG-avid.^24,25^ With invasive ductal cancers, no relationship between ^18^F-FDG uptake in bone metastases and receptor status, including estrogen receptor (ER), Her2 or triple negative breast cancer, has been shown.^24^

There are some data that suggest that in ER+ breast cancers, ^18^F-fluoroestradiol (FES) PET/CT may detect more metastatic lesions (including bone) than ^18^F-FDG PET/CT.^26^ A meta-analysis of four studies that focused on metastatic lesions including bone, showed an overall sensitivity of 78% and specificity of 98% compared to immunohistochemical ER analysis.^27^

High expression of fibroblast activation protein (FAP) is a common feature of many cancers and their metastases, with radiolabeled fibroblast activation protein inhibitors (FAPI), such as ^68^Ga-FAPI PET tracers, showing potential advantages in bone metastasis detection compared to ^18^F-FDG.^28^ Initial preclinical assessment in a breast cancer bone metastasis model has shown higher sensitivity for detection by ^68^Ga-FAPI PET compared to ^18^F-FDG in the early stage of metastasis with the hypothesis that early priming of the metastatic niche by cancer associated fibroblasts allows earlier detection than with ^18^F-FDG. In contrast, ^18^F-FDG becomes more sensitive at a later stage of tumor growth.^29^

Whole-body magnetic resonance imaging (MRI) has become feasible in acceptable acquisition times of less than 1 hour and is therefore more relevant for skeletal staging. In addition to standard morphological sequences including T1-, T2-weighted imaging, and short tau inversion recovery imaging, diffusion-weighted imaging (DW-MRI) is now routine. Morphological sequences allow sensitive detection of disease within the bone marrow and are often more sensitive than bone scans for this reason.^30^ Diffusion-weighted imaging enables sensitive detection of areas of restricted water molecule motion that usually occurs in hyper-cellular tumors and metastases. An increase in water diffusion, as measured by the apparent diffusion coefficient (ADC), occurs after successful therapy as a result of cytotoxicity and reduced cell membrane integrity.^31,32^

### Bone scintigraphy

**Figure 1:**
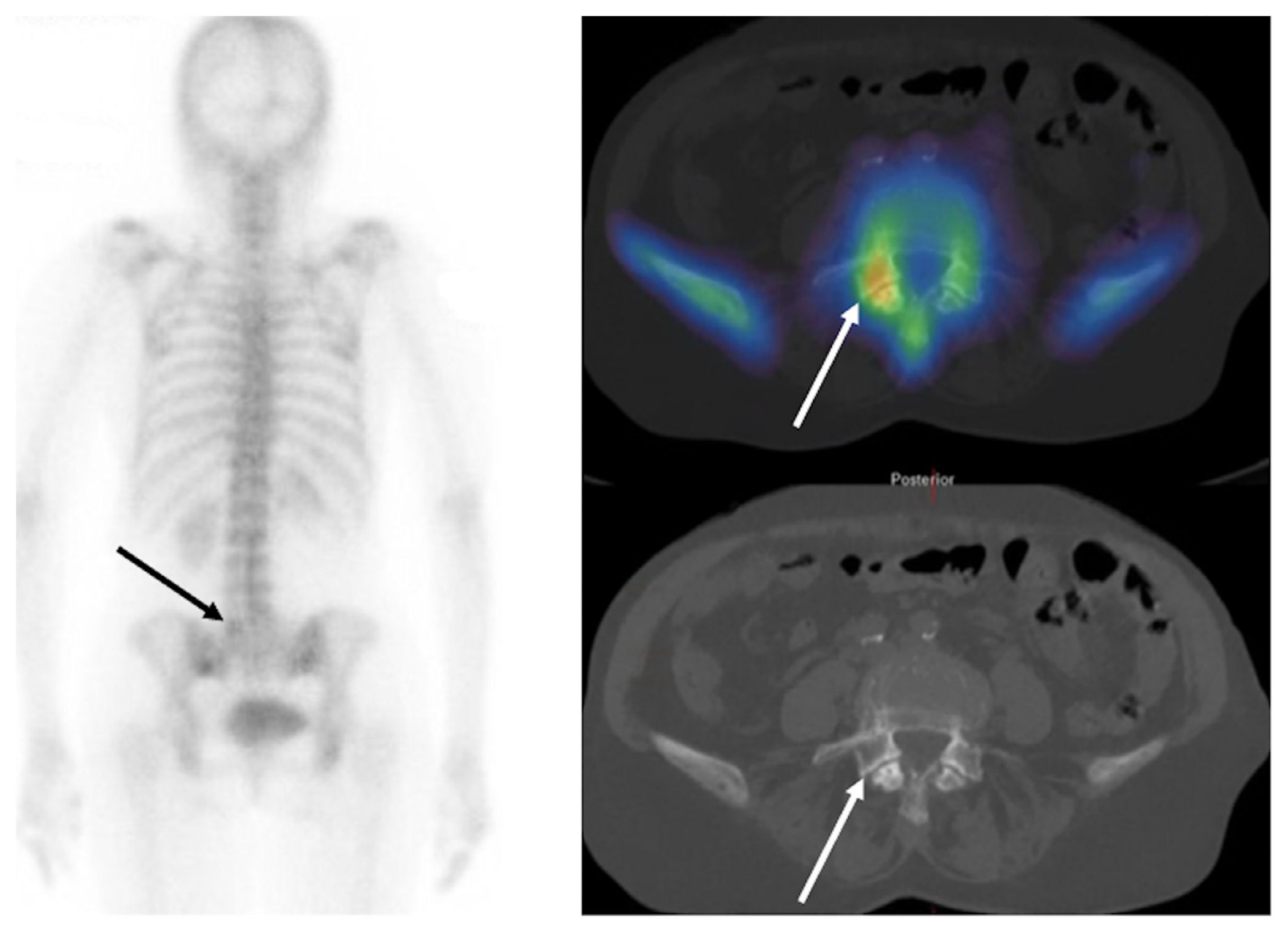
A patient with a new diagnosis of breast cancer and back pain. The planar ^99m^Tc-MDP bone scan (left) shows subtle abnormal uptake in the lower lumbar spine (arrow). SPECT/CT imaging (right) shows the lesion with higher contrast (arrow). The anatomical location on CT (arrow) and morphologic appearances confirm facet joint arthritis illustrating the improvement in specificity that can be gained by the addition of SPECT/CT.

**Figure 2:**
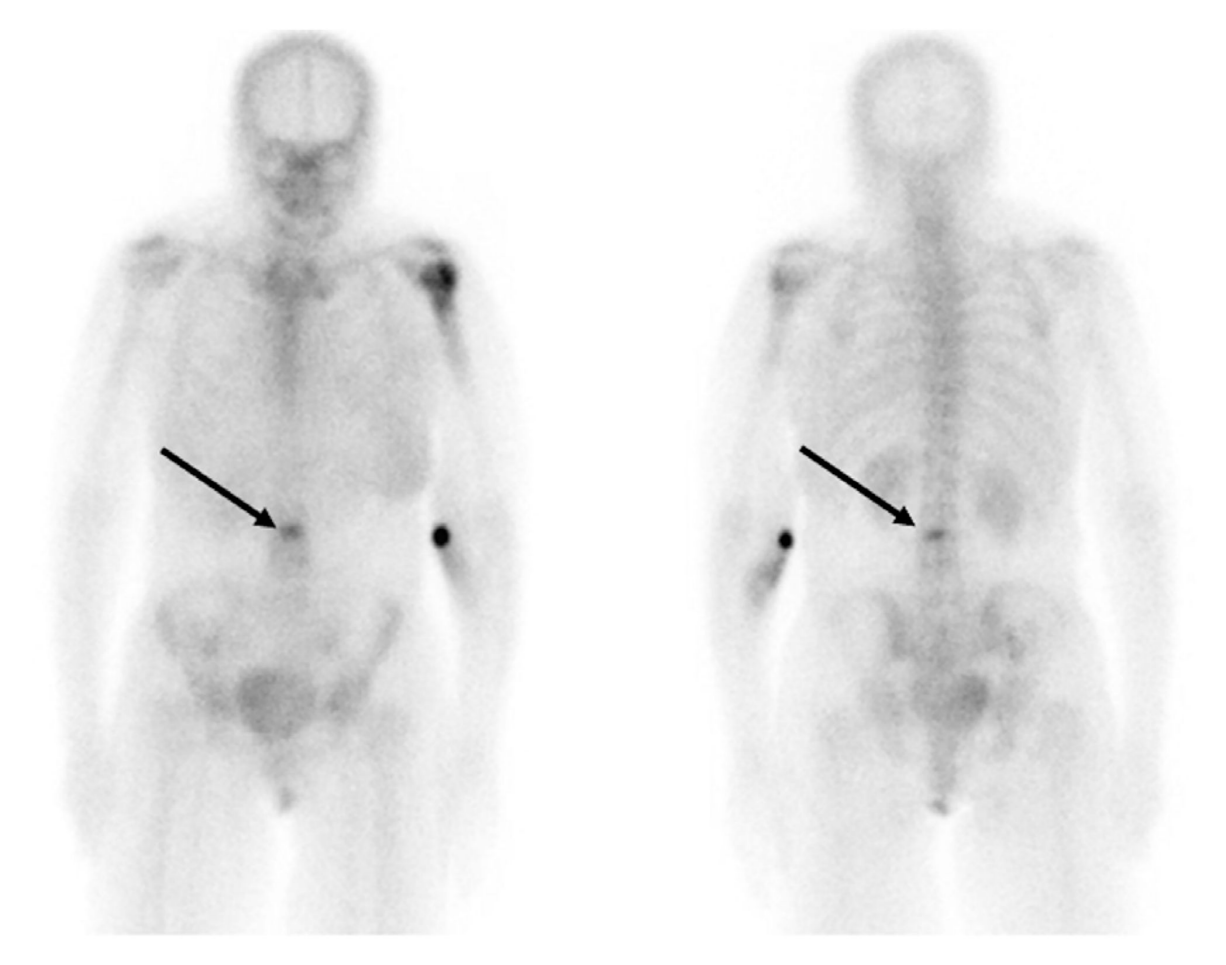

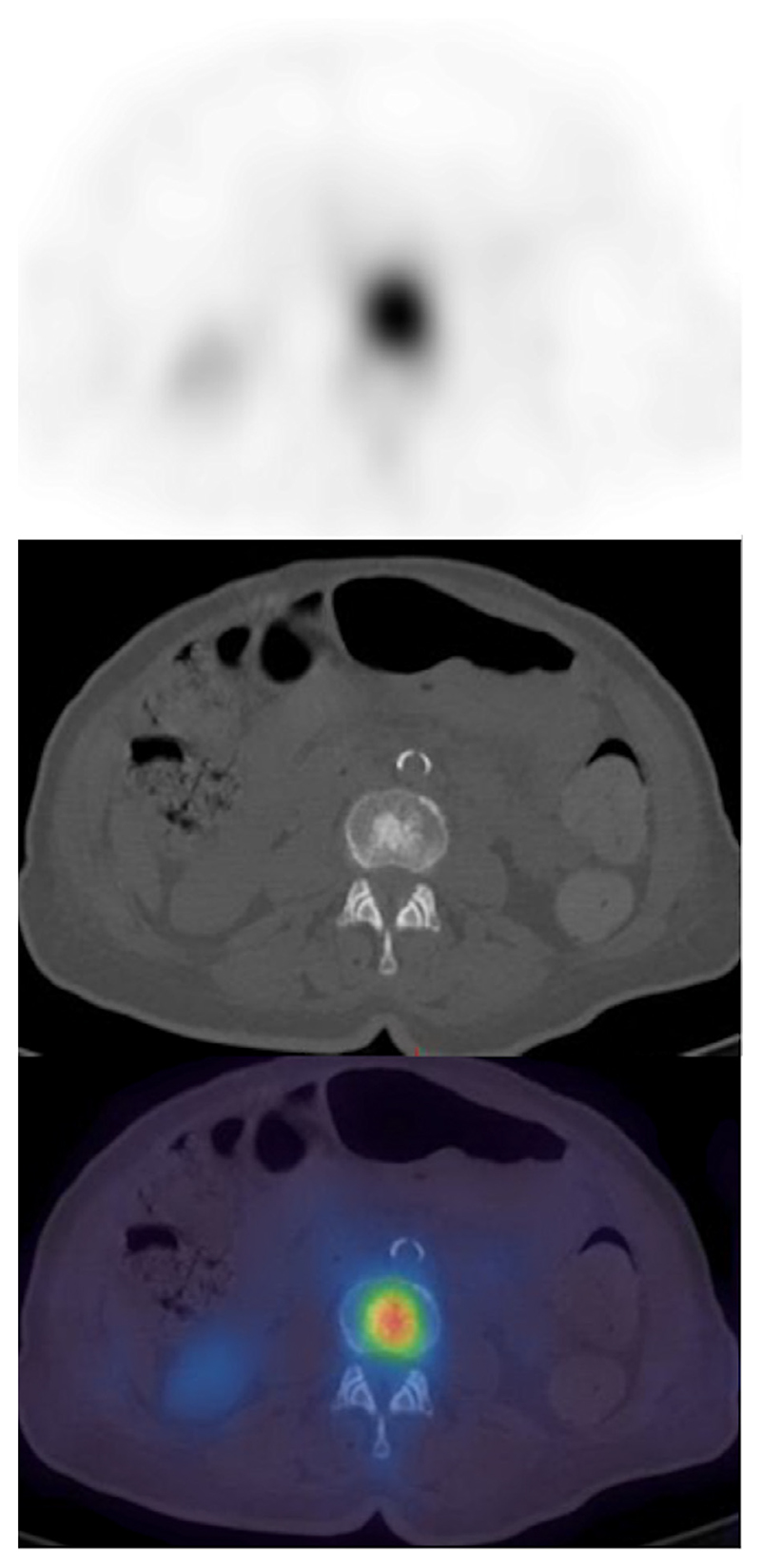
Planar ^99m^Tc-MDP bone scan (a) showing a left humeral metastasis and an indeterminate focus of activity in the mid lumbar spine (arrows). SPECT/CT images (b) localize the abnormal uptake to the vertebral body and show corresponding sclerotic appearances in keeping with a metastasis.

^99m^Tc-labeled diphosphonates show rapid clearance from the blood and soft tissues resulting in optimal skeletal image contrast as early as 2 hours post injection.^33^ Planar bone scintigraphy, using these agents, has been employed for several decades to stage high-risk or symptomatic breast cancer patients and to monitor systemic therapy. Due to recognized limitations with sensitivity and specificity, it is now less frequently used. However, the improvements afforded by single photon emission computed tomography (SPECT) imaging through increased contrast and better spatial localization of lesions, further complemented by the addition of SPECT/CT in more recent years, has optimized detection and characterization of focal tracer accumulation,^34,35^ giving the bone scan a new lease of life in competition with the other evolving modalities used in this indication (Figs. 1 and 2). Nevertheless, the indirect detection of tumor in the skeleton with bone-specific tracers has some limitations that the addition of SPECT or SPECT/CT will not necessarily overcome. Early marrow lesions, before an osteoblastic reaction has occurred, or lesions that are predominantly lytic without a significant osteoblastic component, will have limited sensitivity for detection. In addition, there are limitations in sensitivity and specificity for evaluating systemic treatment response of bone metastases from breast cancer. In an early study, only 52% of responding patients showed improvement on the bone scan and only 62% of nonresponders showed scintigraphic deterioration after 6-8 months of treatment^36^. Response assessment is also hampered by the flare phenomenon where it may take several weeks, or as long as 6 months, to be able to differentiate progressive disease from osteoblastic healing after successful therapy.^16,17^

It is recognized that conventional methods for measuring response to therapy in tumors are limited for bone metastasis assessment. The Response Evaluation Criteria in Solid Tumors (RECIST) criteria are insufficient for the majority of bone metastases as only lytic disease with associated soft tissue masses >10 mm are considered suitable for response evaluation.^37^ In spite of this, the MD Anderson criteria were developed for patients with bone-predominant breast cancer using a combination of radiographs, bone scan, CT and MRI. These criteria, when comparing responders to non-responders, were able to predict progression-free survival (PFS) better than WHO response classification criteria but not in terms of overall survival (OS).^38^

### ^18^F-fluoride PET

^18^F-fluoride was first described as a bone tracer in the 1960s^39^ but it was not until the advent of modern PET and PET/CT scanners that it was possible to take advantage of some of the superior properties of this bone-specific PET tracer. Like the ^99m^Tc-labeled diphosphonates, the mechanism of uptake in metastases reflects local blood flow and increased osteoblastic activity. However, some of the pharmacokinetics are superior in that it has near 100% first pass extraction in bone, negligible protein binding and rapid renal excretion. Combined with the superior spatial resolution of PET, these properties allow high resolution and high contrast imaging at less than 1 hour post injection.^14^ These factors contribute to the superior diagnostic accuracy of ^18^F-fluoride PET/CT compared to bone scintigraphy ± SPECT/CT that has been reported.^40–42^

A National Oncology PET Registry trial assessed the impact of ^18^F-fluoride PET/CT on the management of patients with cancer other than prostate cancer and included 781 patients with breast cancer. Clinical management changed in 24% for those with a suspected first bone metastasis and in 60% for with suspected progression of bone metastases.^43^

**Figure 3:**
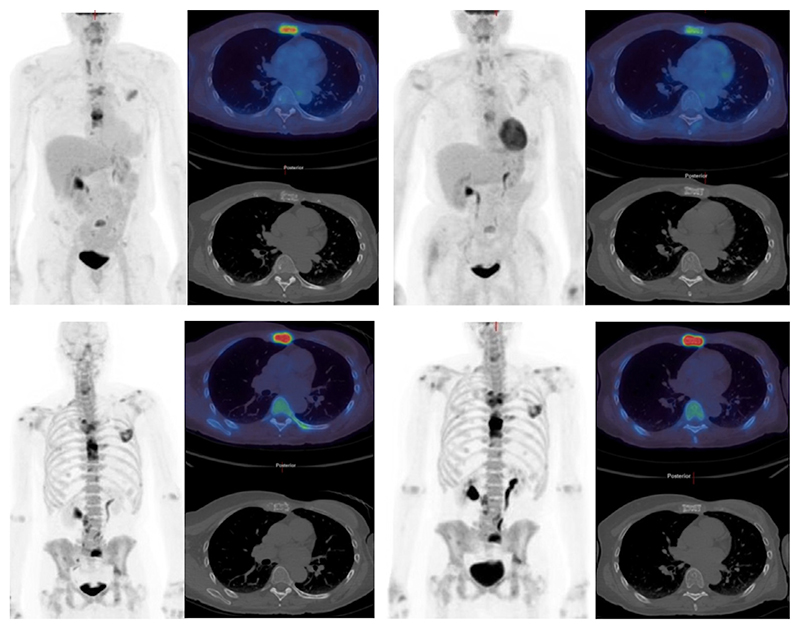
(a) ^18^F-FDG PET/CT maximum intensity projection image (left) and axial (right) images showing increased ^18^F-FDG uptake in a sternal metastasis with mixed lytic and sclerotic appearances on CT. After 8 weeks of endocrine treatment the corresponding images (right) show a reduction in uptake (SUVmax decreased from 11.5 to 5.3) confirming a partial metabolic response. (b) ^18^F-fluoride PET/CT images at the same time points showing an increase in activity in the sternal metastasis (SUVmax increased from 37.3 to 68.3) due to the flare phenomenon.

There are limited data exploring the efficacy of ^18^F-fluoride PET/CT in monitoring treatment response. In a small comparative prospective study of endocrine treatment response in 22 patients with bone-predominant metastatic breast cancer, changes in maximum standardized uptake value (SUVmax) between baseline and 8-week ^18^F-fluoride PET predicted progressive disease with 60% sensitivity, 73% specificity, and 70% accuracy. Seven of 20 patients showed a flare defined as an initial increase in SUVmax that subsequently declined by 12 weeks^44^ (Fig. 3).

Feasibility of dynamic ^18^F-fluoride PET measurement of kinetic indices, including fluoride transport (K1) and flux (Ki) has been shown for evaluation of response in breast cancer metastases.^45^ Another study, employing a simplified method for calculating Ki from static scans, and a population input function corrected by venous blood samples, showed that changes in Ki after 8 weeks of endocrine treatment were able to more reliably differentiate those with progressive disease compared to changes in SUVmax.^46^ In clinical practice, a further National Oncology PET registry trial of 476 patients with breast cancer investigating ^18^F-fluoride PET/CT for treatment evaluation led to a change in clinical management plans in 39%.^47^

### ^18^F-Fluorodeoxyglucose PET/CT

**Figure 4:**
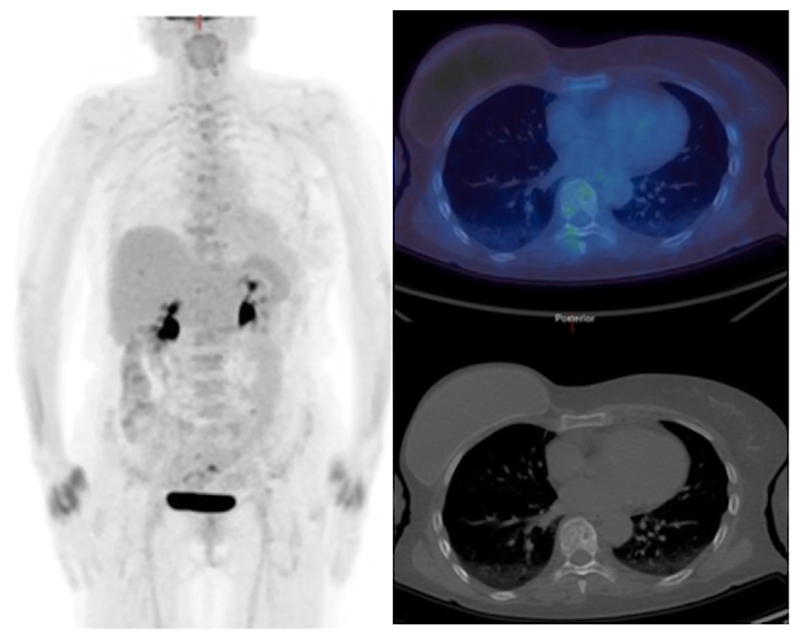

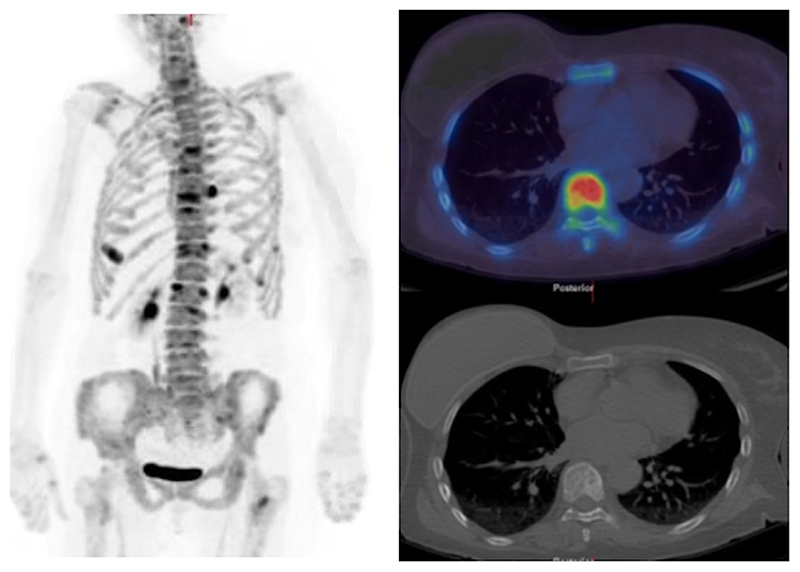
A woman with metastatic breast cancer. The ^18^F-FDG PET/CT scan (a) (maximum intensity projection image - left, axial images through a thoracic vertebra - right) shows only low-grade accumulation in sclerotic metastatic disease. The corresponding ^18^F-fluoride PET/CT scan (b) shows much higher activity reflecting the osteoblastic nature of the metastatic disease in this patient.

Bone metastasis phenotype appears to be related to ^18^F-FDG avidity in breast cancer. Patients with untreated or progressive sclerotic metastases show lower uptake and better survival than those with metastatic disease that is predominantly lytic in nature^24,25,48^ (Fig. 4). This observation may be partly due to the inherent lower glucose metabolism seen in lobular breast cancers, a subtype often associated with primarily sclerotic disease, and partly that osteoblastic metastases may have relatively small tumor volume compared to the accompanying osteoblastic reaction in bone. Successfully treated bone metastases that show reductions in ^18^F-FDG uptake frequently show increased sclerosis on radiographs or CT, even when not primarily osteoblastic in nature, reflecting the osteoblastic repair mechanism. A treatment history is therefore essential to interpret the metabolic and morphologic appearances of bone metastases in ^18^F-FDG PET/CT.^49^ There are several reports and meta-analyses that confirm ^18^F-FDG PET/CT is more sensitive and specific for detecting bone metastases in breast cancer, particularly when lytic metastases predominate.^50–52^ This may reflect the ability of ^18^F-FDG to directly assess tumor metabolism rather than a reaction in the bone microenvironment before there is an osteoblastic reaction, as required for bone-specific tracers.

**Figure 5:**
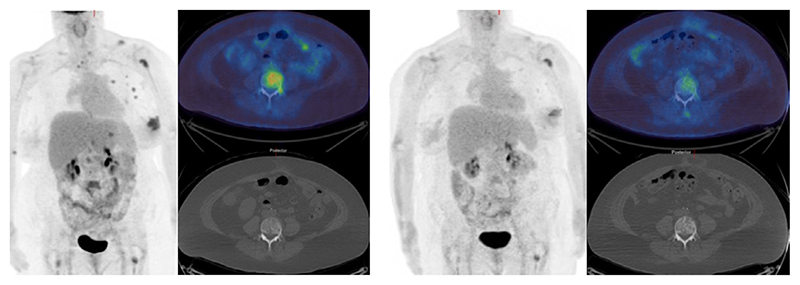
^18^F-FDG PET/CT (maximum intensity projection image - left, axial images through L4 - right) show high uptake in a primary left breast cancer and bone metastases with some underlying sclerosis on the CT component. After 8 weeks of endocrine therapy a partial metabolic response is visible (L4 metastasis SUVmax decreased from 8.4 to 4.4).

There are increasing data confirming the ability for changes in ^18^F-FDG uptake (usually measured by SUVmax) to predict and monitor systemic treatment response of bone metastases (Fig. 5). In a retrospective study of 102 patients, both reduction in SUVmax and increase in sclerosis on CT were predictors of response duration on univariate analysis but on multivariate analysis only reduction in SUVmax remained predictive.^53^ Other studies have shown SUVmax changes correlate with clinical and tumor marker response assessment^54^ and are predictive of time to progression or time to SREs.^55^ A correlation between response or progressive disease on ^18^F-FDG PET with circulating tumor cell counts has also been observed.^56^ A prospective study of 22 patients, 15 of whom had bone metastases, on endocrine therapy for metastatic breast cancer performed ^18^F-FDG PET/CT at baseline and 10 (±4) weeks and used changes in SUVmax for response measurements according to EORTC PET response criteria.^57^ A statistically significant difference in PFS but not OS was found between those with progressive metabolic disease and non-progressors (either responders and/ or stable disease).^58^ In a further prospective study of patients being treated with endocrine therapy for bone-pre-dominant metastatic breast cancer, changes in ^18^F-FDG SUV-max predicted non-progression and PFS at 6 months.^44^ This study also reported heterogeneity of response between metastases in individual patients. Another prospective study of 28 patients reported that changes in ^18^F-FDG uptake in bone metastases at 4 months were able to predict time to SREs and time to progression but not OS. Of interest is that ^18^F-fluoride PET changes in the same cohort of patients did not predict these parameters but an increase in ^18^F-fluoride SUVmax did predict OS, probably reflecting the flare phenomenon in responding metastases.^59^

### Other PET tracers

^18^F-fluoroestradiol targets the ER which is frequently overexpressed in breast cancer and usually assessed by immunohistochemical analysis of biopsy material to determine suitability for endocrine therapy. The potential weakness of a single biopsy to measure heterogeneity of ER between metastases was highlighted by a study of breast cancer bone metastases using both ^18^F-FDG and ^18^F-FES. In patients treated with endocrine therapy, it was reported that ^18^F-FDG predicted PFS but only heterogeneity of ER expression, measured by the ratio of tumor burden between ^18^F-FES and ^18^F-FDG scans, was able to predict OS. Patients with a low ratio had a worse prognosis.^60^ Further studies of ^18^F-FES in metastatic breast cancer noted heterogeneity between different metastatic sites, with bone metastases showing higher uptake than lymph node or lung metastases,^61^ and significant heterogeneity within and between individual patients.^62^

^68^Ga-FAPI tracers have shown high uptake in a variety of different tumors and there is also some early evidence that this tracer may be beneficial in breast cancer with primary tumors as well as metastases at most metastatic sites (including bone) showing higher uptake and sensitivity compared to ^18^F-FDG.^63,64^ The mean SUVmax in bone lesions for ^68^Ga-FAPI was 7.3 compared to 4.9 for ^18^F-FDG and in the 17 patients with bone lesions, ^68^Ga-FAPI detected 205 bone metastases while ^18^F-FDG detected 146.

## Whole-body and diffusion-weighted MRI

**Figure 6:**
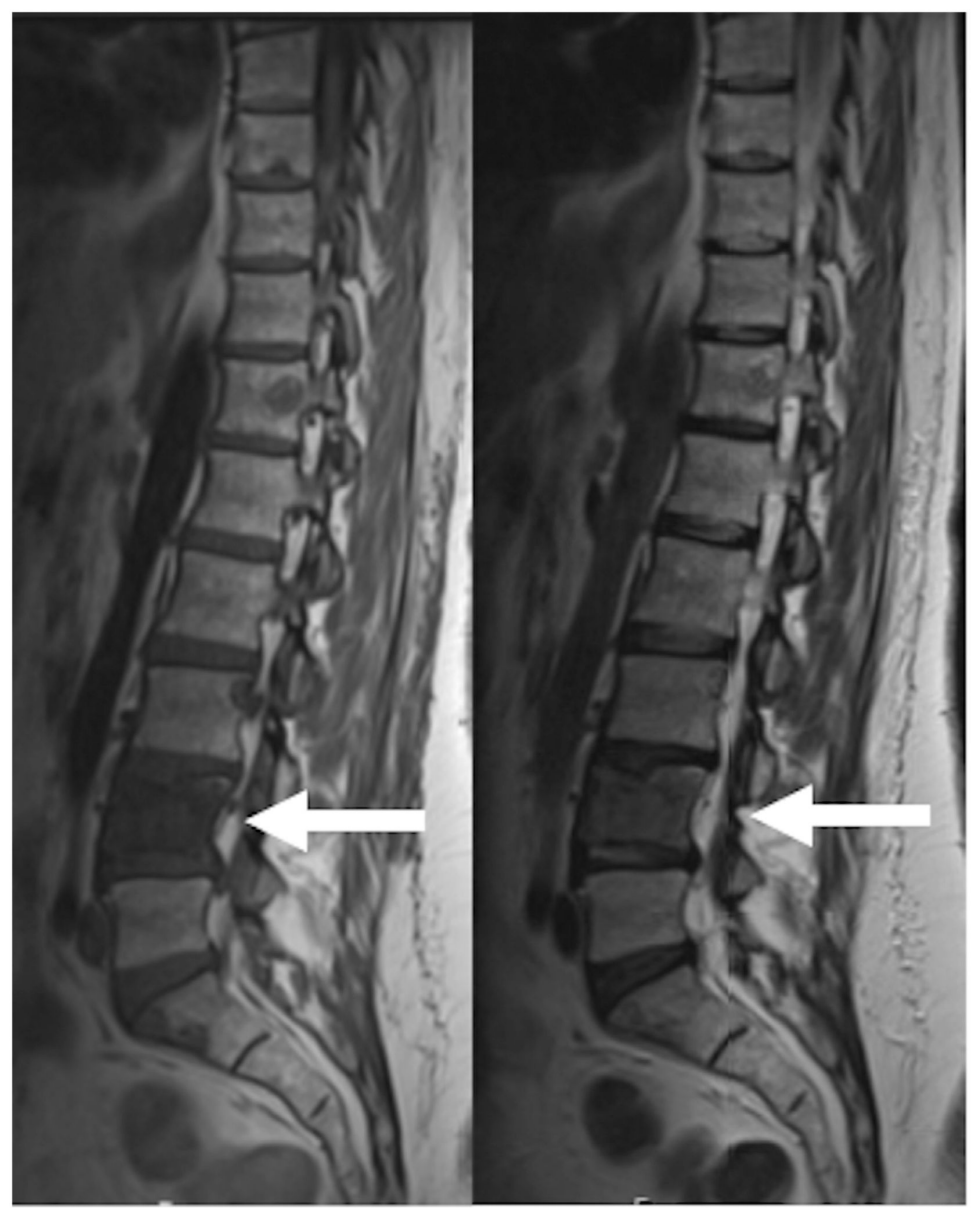

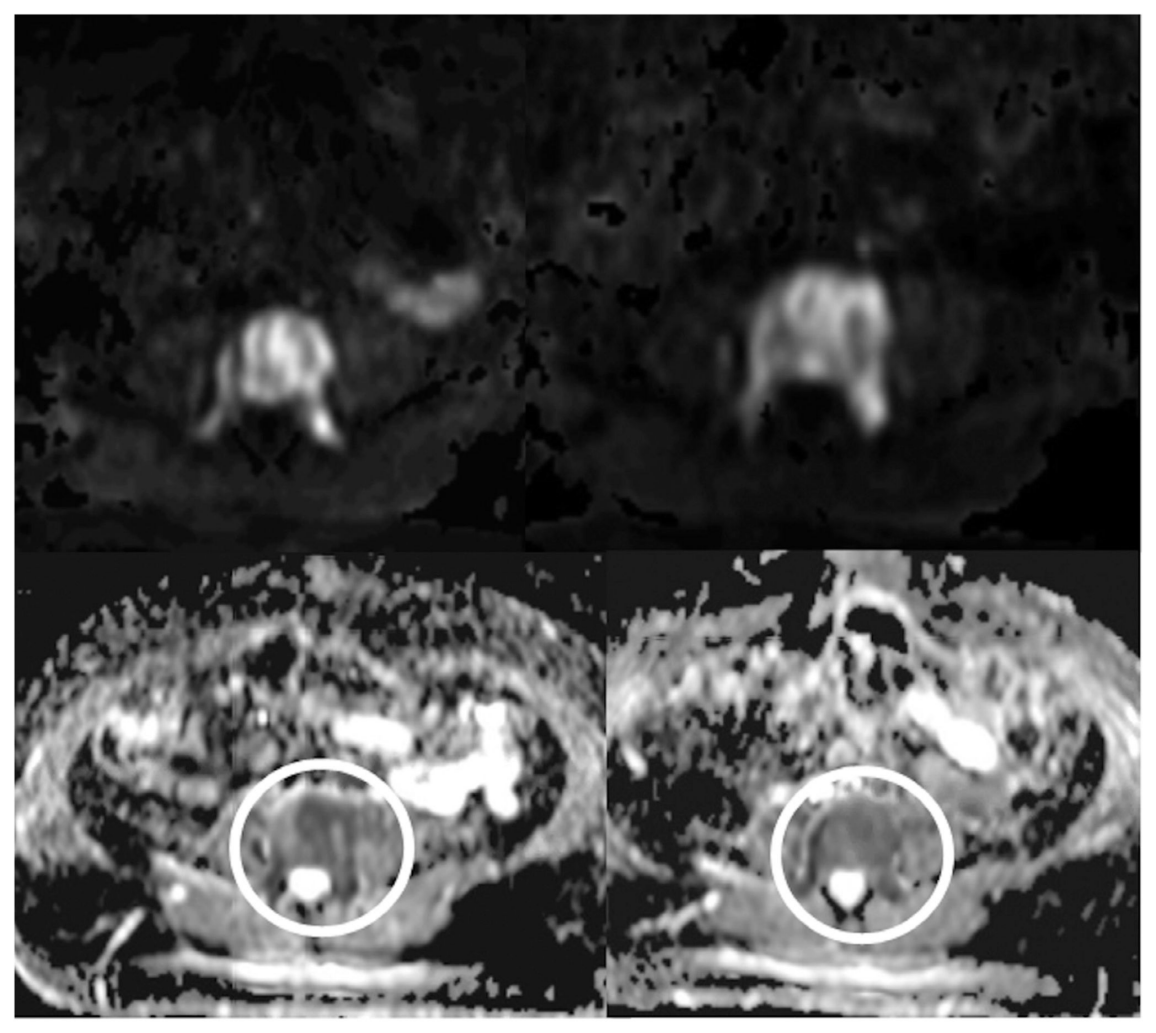
The same patient as Figure. 5. (a) T1-weighted (left) and T2-weighted (right) sagittal MRI of the spine showing low signal in L4 due to metastatic marrow infiltration (arrows). (b) Top row - axial b900 DWI at baseline and 8 weeks after therapy showing high signal in L4. The ADC map (bottom row) shows evidence of restricted diffusion in the vertebra (circles). The ADC increased from 900 to 1108 mm^2^/s in keeping with treatment response.

Conventional spin-echo MRI sequences return poor signal from bone but can readily demonstrate bone marrow. The standard morphological MRI sequences detect altered signal in bone marrow at sites of skeletal metastases due to differences in proton density (water content) between tumor and normal marrow. Metastases typically return a lower signal than bone marrow on T1- and T2-weighted imaging. On diffusion-weighted imaging, the b-value measures the degree of diffusion weighting applied and reflects the strength of the diffusion effects. Metastases will appear of higher signal on increasing b-value sequences and of higher ADC than the normal marrow due to differences in cell size and distribution compared to normal fat cells in marrow (Fig. 6). The functional DW-MRI images are not read in isolation, requiring correlation with morphologic sequences. A meta-analysis of studies with bone metastases from varied cancers has confirmed high sensitivity (90%) and specificity (92%) for whole-body MRI with diffusion-weighted imaging but with a lower specificity in the studies where DW-MRI was included suggesting that false positives may be occasionally introduced by DW-MRI.^65^

A prospective study compared bone scan (±SPECT/CT), ^18^F-fluoride PET/CT and whole-body DW-MRI. Whole-body DW-MRI showed similar diagnostic accuracy to ^18^F-fluoride PET/CT (91% and 93% sensitivity, respectively) for detecting breast cancer bone metastases.^66^ Diffusion-weighted MRI has also been reported to be as sensitive as ^18^F-FDG PET/CT in metastatic breast cancer, but less specific, particularly in lymph nodes and the skeleton.^67^

A comparison of ^18^F-FDG PET/CT with PET/MRI in malignant skeletal disease, including 19 patients with breast cancer, reported similar lesion conspicuity and classification on PET but better anatomical delineation on MRI compared to CT.^68^

## Conclusions

Bone metastases from breast cancer are common and are responsible for significant morbidity and healthcare costs. With the advent of novel, more effective therapies early detection and accurate response assessment has become even more important. Molecular and hybrid imaging techniques have led to advances in the field and improvements in patient management. However, there is no clear guidance or evidence of superiority on one imaging method over another and choice will depend on local costs, expertise and availability at each institution.
